# Hearables: New Perspectives and Pitfalls of In-Ear Devices for Physiological Monitoring. A Scoping Review

**DOI:** 10.3389/fphys.2020.568886

**Published:** 2020-10-16

**Authors:** Michela Masè, Alessandro Micarelli, Giacomo Strapazzon

**Affiliations:** ^1^Institute of Mountain Emergency Medicine, Eurac Research, Bolzano, Italy; ^2^Healthcare Research and Innovation Program, IRCS-HTA, Bruno Kessler Foundation, Trento, Italy; ^3^ITER Center for Balance and Rehabilitation Research (ICBRR), Rome, Italy

**Keywords:** wearables, earbuds, physiological monitoring, temperature, oxygen saturation, heart rate, hypothermia, heat exercise

## Abstract

Technological advancements are opening the possibility of prolonged monitoring of physiological parameters under daily-life conditions, with potential applications in sport science and medicine, and in extreme environments. Among emerging wearable technologies, in-ear devices or hearables possess technical advantages for long-term monitoring, such as non-invasivity, unobtrusivity, good fixing, and reduced motion artifacts, as well as physiological advantages related to the proximity of the ear to the body trunk and the shared vasculature between the ear and the brain. The present scoping review was aimed at identifying and synthesizing the available evidence on the use and performance of in-ear monitoring of physiological parameters, with focus on applications in sport science, sport medicine, occupational medicine, and extreme environment settings. Pubmed, Scopus, and Web of Science electronic databases were systematically searched to identify studies conducted in the last 10 years and addressing the measurement of three main physiological parameters (temperature, heart rate, and oxygen saturation) in healthy subjects. Thirty-nine studies were identified, 24 performing temperature measurement, 12 studies on heart/pulse rate, and three studies on oxygen saturation. The collected evidence supports the premise of in-ear sensors as an innovative and unobtrusive way for physiological monitoring during daily-life and physical activity, but further research and technological advancement are necessary to ameliorate measurement accuracy especially in more challenging scenarios.

## Introduction

Physiological parameter trackers are an evolving technology that allow to monitor and collect physiological data, such as heart rate (HR), temperature (T), oxygen saturation (SpO_2_), and energy consumption (Bunn et al., 2019). These wearables are conceived for multiple uses from bench-to-bedside to daily-life conditions, and for recreational, clinical, and research purposes (Poh and Kittler, 2012; Leboeuf et al., 2014; Skaiaa et al., 2015; Strapazzon et al., 2015; Budidha and Kyriacou, 2018; Bunn et al., 2019). Wearables are appealing technologies in fields, such as sport science, sport medicine, occupational medicine, and other out-of-hospital medical situations, where device portability and unobtrusivity may represent key advantages.

To satisfy these settings, a device must be accurate and reliable under multiple environmental and activity conditions, and it should be also lightweight, easy to wear and handle, minimally invasive, and discrete to favor long-term monitoring (Leboeuf et al., 2014). Additional requirements may be needed to extend the use of these devices to settings with challenging environmental conditions. Since several physiological parameters are estimated by devices relying on photo-plethysmography (PPT, measurement of light absorbance/reflectance from the vascular bed) from different peripheral body areas, environmental conditions that seriously impact perfusion may compromise the reliability of measurements. To overcome these restrictions, the application of sensors on better-perfused areas has been proposed (Rosenberg and Pedersen, 1990; Clayton et al., 1991; Kyriacou, 2006; Budidha and Kyriacou, 2018). However, application sites on the skin may experience functional difficulties, such as attachment problems and motion artifacts (Clayton et al., 1991; Budidha and Kyriacou, 2018), while sites within body cavities (naso-pharyngeal, gastro-intestinal, etc.) may limit general applicability due to the expertise required for application (semi-invasivity) and potential discomfort.

The ear canal has been proposed as a promising measurement site for physiological parameters, potentially able to combine minimal invasivity and wearability with reliable and accurate recordings in different settings (Poh and Kittler, 2012; Budidha and Kyriacou, 2014; Leboeuf et al., 2014; Bunn et al., 2019). Thanks to its close position to the central nervous system and major vasculature, it can provide better signal quality and more stability. The area is slightly influenced by the sympathetic nerve activity in conditions leading to low perfusion states, resulting in an adequate blood flow, higher quality of PPT signals, and reliable pulse rate (PR) and SpO_2_ monitoring. The site provides good fixation and unobtrusivity, facilitating long-term monitoring in daily-life conditions, and it offers protection from challenging environmental conditions (low temperature, sunlight) for in-field applications in challenging conditions (Budidha and Kyriacou, 2014, 2015).

In this context, the aim of the present scoping review was to identify and summarize the studies conducted in the last 10 years on the measurements of physiological parameters from the human ear canal in healthy subjects. In particular, the search was focused on the monitoring of three basic parameters, such as T, HR/PR, and SpO_2_, in settings related to sport science and occupational medicine, including challenging environmental scenarios where such measurements may be hindered. The structure of the scoping review is the following. Sections Ear Anatomy and Vascularization and Measurement of Physiological Parameters From the Ear Canal provide basic introductory concepts on ear anatomy and vasculature and on the techniques applied for physiological monitoring from the ear canal. Section Scoping Review Methods describes the search strategy and data extraction process. Section Results summarizes the studies identified by the systematic search for the three main outcomes (i.e., T, HR/PR, and SpO_2_). For each parameter, a brief introduction of its importance in the field of sport science/medicine and occupational medicine is provided, together with the selective advantages of in-ear measurement in that context. Finally, Section Limitations and Future Developments provides a general discussion about potential limitations and pitfalls of hearables, as well as future challenges and perspectives of such technology.

## Ear Anatomy and Vascularization

**Figure 1:**
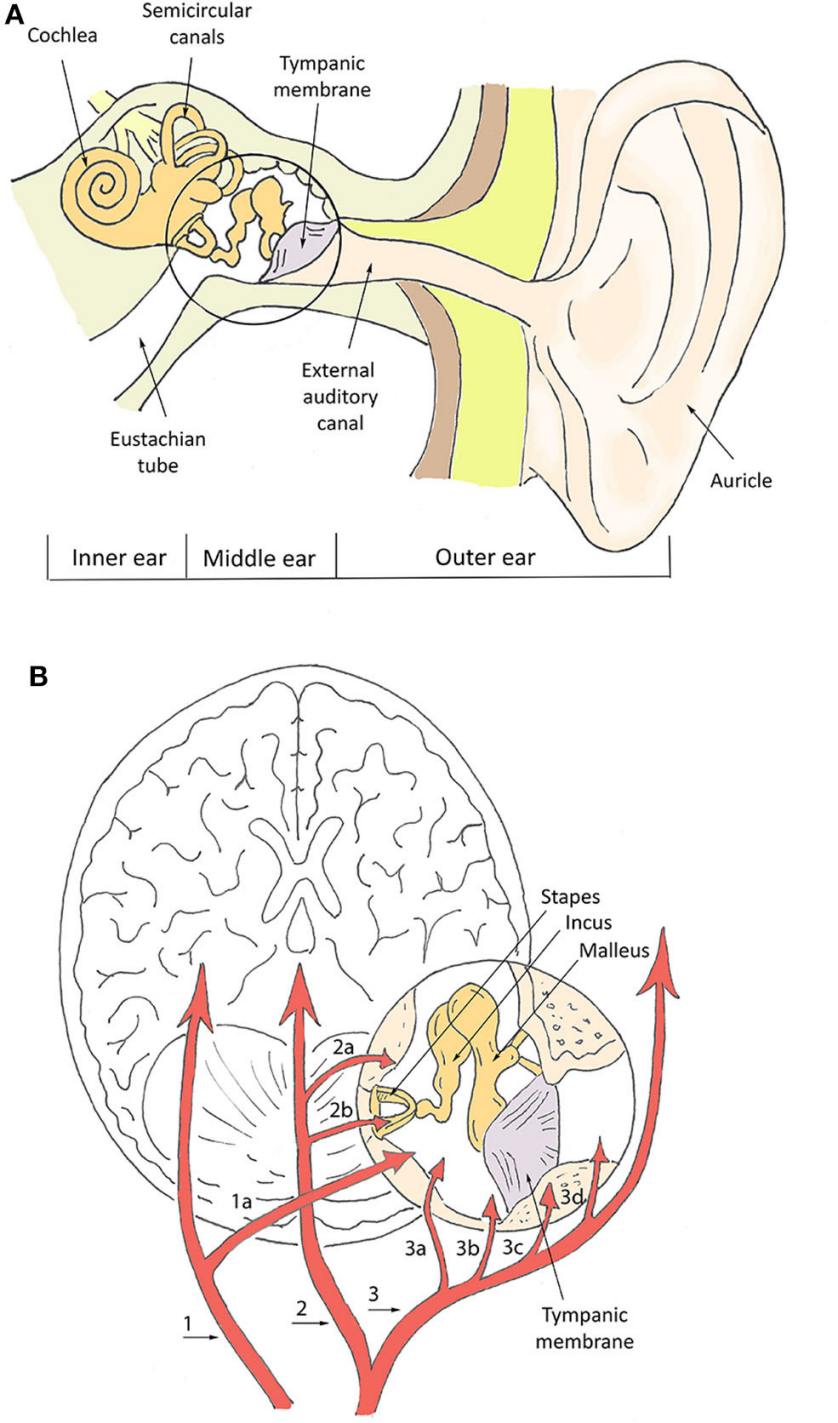
Schematization of ear canal anatomy **(A)** and vasculature **(B)**. In **(B)**, numbers indicate the main vessels and specifically: the basilar artery (1), internal auditory artery (1a), internal carotid artery (2), artery of pterygoid canal (2a), carotico-tympanic artery (2b), external carotid artery (3), maxillary artery (3a), stylomastoid artery (3b), posterior auricular artery (3c), anterior tympanic artery (3d).

The measurement of physiological parameters from the ear canal in proximity of the tympanic membrane (TM) is strictly related to the characteristic vasculature of this area. The basic aspects of ear anatomy and vasculature are schematized in Figure 1. In terms of vasculature (Figure 1B), the brain and the TM are both supplied by the basilar artery and the internal carotid artery (Benzinger and Taylor, 1963; McCarthy and Heusch, 2006). The basilar artery supplies the TM via the internal auditory artery. The internal carotid artery provides blood to the TM via the artery of the pterygoid canal and the carotico-tympanic branch, which have anastomoses with a vascular circle formed by several branches of the external carotid artery, such as the anterior tympanic artery, posterior auricular artery, stylomastoid artery, and maxillary artery (Berkovitz, 2005; McCarthy and Heusch, 2006). The anastomoses with branches of the internal carotid arteries are crucial for monitoring physiological parameters. The internal carotid arteries (one on each side of the head) are the main blood supply to the circle of Willis and to the brain, including crucial sites for T regulation like the hypothalamus. In-ear measurement of core T assumes that the TM is supplied by blood from the same sources that supply the brain, which guarantees thermal equilibrium between the two sites. The measurement of SpO_2_ and PR mostly relies on the acquisition of PPT signals, whose quality is also related to blood supply. Ear vascularization can guarantee adequate flow even in pathophysiological situations, such as in accidentally hypothermic patients where even cerebral autoregulation is progressively lost (Paal et al., 2018; Gaasch et al., 2020).

On the other hand, given the frailty of the TM, some conditions affecting its integrity have been hypothesized to impact in-ear physiological measurements (Tasli and Gökgöz, 2018). Several studies showed that pathological conditions, such as acute external otitis, cerumen obturans, and previous major ear surgery may significantly alter the measurement of core T (García Callejo et al., 2004; Schmäl et al., 2006; Tasli and Gökgöz, 2018). Differently, small perforation (<10 mm) in the central low-irrorated area of the TM, otitis media, fluid in the middle ear, ventilation tubes, and minor surgery seem to have negligible effects (García Callejo et al., 2004; Schmäl et al., 2006; Tasli and Gökgöz, 2018).

## Measurement of Physiological Parameters From the Ear Canal

**Figure 2:**
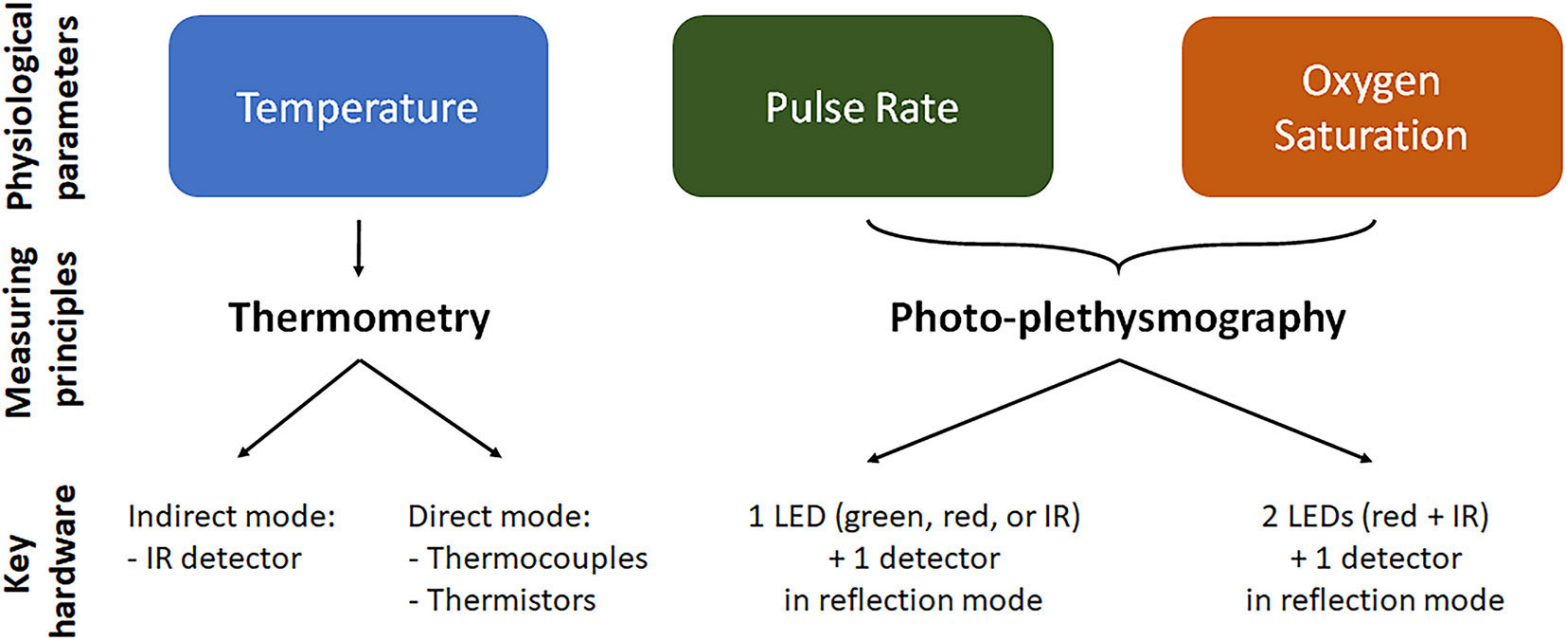
Physiological parameter measurement in the ear canal. For each of the parameter we reported the mainly used measurement technique and the key elements/working configuration of the measuring device. Temperature is measured by thermometry approach using indirect infrared (IR) thermometers or direct thermopile or thermocouple thermometers. Oxygen saturation and pulse rate are measured using photo-plethysmography, where light is emitted at two or one wavelength, respectively, and the reflected light is detected and analyzed. LED, light emitting diode.

The monitoring of T, HR/PR, and SpO_2_ from the ear canal relies on different measuring principles and devices, which are briefly summarized in Figure 2. T measurement relies on thermometric approaches, which work in direct or indirect mode. In direct mode thermometers, the output T corresponds to the T of the sensor, which is thermally coupled to the measuring site (Ring et al., 2010). Thermistor-based thermometers work in direct mode and utilize the intrinsic property of materials, such as metallic oxides, to change their electrical resistance as a function of T. Thermistor-based thermometers include a metallic probe, usually encapsulated in an impermeable material, such as a soft rubber earplug, an electronic circuitry to measure the resistance change and a microprocessor for calibration and data display (Chen, 2019). Potential limitations of direct approaches may include a slow time response to reach thermal equilibrium with the measuring site, and difficulties in placing the sensor close to the desired body site. In indirect or adjust-mode thermometers, the output T is the result of a signal adjustment or conversion, based on clinical data and physiological and anatomical properties, which corrects for differences between the measuring and sensor sites. The use of signal processing tools to estimate T results in fast response time, but may reduce actual accuracy (Ring et al., 2010). Infrared (IR) thermometers, the most widespread methodology to measure tympanic T, are adjusted-mode thermometers, based on the detection of the IR radiation emitted by the TM and its conversion to an electrical signal. IR tympanic thermometers (IRTT) are usually composed of a probe tip with lenses to focus the IR light, a sensing electronic module (thermopile or pyroelectric sensor) for signal transduction, and a microprocessor circuitry for data calibration and display (Chen, 2019). Although standard requirements for IRTT recommend clinical accuracy of ±0.3°C for target T in the range 33–42°C at ambient temperatures of 16–33°C (ISO 80601-2-56, 2017), actual accuracy in the clinical setting may be affected by several factors, related—but not limited—to patient variability (gender, age, thermoregulation, handedness), local phenomena (excessive cerumen, ear major infection or surgery, tissue cooling due to repeated measurements), operator's experience, device maintenance, and environmental conditions (humidity, temperature). The latter may particularly affect accuracy during in-field applications (Ring et al., 2010; Sund-Levander and Grodzinsky, 2013).

The measurement of PR and SpO_2_ from the ear canal is mainly based on optical sensing techniques, although also piezo-electric sensors and IR thermography techniques have been proposed for PR measurements. In optical techniques, a peripheral site with high vascular density is illuminated by a light source and the light reflected by the vascular bed (PPG signal) is measured by a photo-detector (see Figure 2). The PPG signal is composed by a direct constant component, which includes absorption/reflectance due to arterial blood, venous blood, and capillaries, and by a pulsatile alternating component, which is synchronous with the heart beat and is representative of tissue perfusion (Foo et al., 2013). The pulsatile component can be directly used to estimate PR. Devices for in-ear PR measurement include as principal elements one light emitting diode (LED) for light emission at a single wavelength (red, IR, or green), one photo-detector to measure the reflected light, and a microprocessor to control the LED and calculate PR values (Poh et al., 2012). PR measurements using red or IR light can be affected by IR rays in sunlight, preventing stable operation for outdoors applications, thus indoors or semi-indoors usage is recommended. For outdoors PR measurement, a green light source is usually preferred, being less susceptibility to ambient light (Lemay et al., 2014; Parak, 2018).

With respect to PR, the measurement of SpO_2_ requires light emission and detection at two or more different wavelengths. SpO_2_ is defined as the fraction of oxyhemoglobin (i.e., hemoglobin bound with oxygen) to deoxyhemoglobin (i.e., hemoglobin not bound with oxygen), which can be optically distinguished by their distinct light absorbance/reflectance at different wavelengths (i.e., oxyhemoglobin absorbs greater amounts of IR and lower amounts of red light than deoxyhemoglobin). The simplest but conventional approach to measure SpO_2_ is to analyze the light of the two wavelengths reflected from the pulsatile added volume of the oxygenated arterial blood and, specifically, calculating the absorption rate given by the double ratio of the pulsatile and non-pulsatile components of red light to IR light reaching the photo-detector (Foo et al., 2013). Devices to measure SpO_2_ include two LEDs, emitting red and IR light, a photo-detector to measure the reflected light, and a microprocessor controlling led switching and converting absorption rate values into SpO_2_ values.

Standard requirements for optical assessment of physiological parameters (ISO 80601-2-61, 2017) recommend that PR accuracy is of ±3% in the clinical range 40–240 bpm, while SpO_2_ accuracy is ±2% in the clinical range 84–100% and ±3% in the clinical range 70–100%. Nevertheless, technical and physiological factors may interfere with optical measurements affecting accuracy. These factors include motion artifact from physical movement, misalignment/distance between the skin and the optical sensor, geometry of the sensor, variation in the intensity and spectrum of the light source, ambient light, variations in skin color/tone, size, and depth of the vascular area, poor tissue perfusion (Lemay et al., 2014; Parak, 2018; Bent et al., 2020). In particular, working in reflection mode, in-ear devices may be affected by high shunt light (i.e., the amount of direct light traveling from the LED to the detector without propagation over the pulsing blood in the biological tissue) and to the lower signal amplitude with respect to transmission mode signals (Budidha and Kyriacou, 2014; Lemay et al., 2014; Parak, 2018). To improve PPG signals, optimal designing of sensor geometry and LED-photodetector distance based on the used wavelength is crucial, but also alternative sensor configurations, such as “circummission” mode have been proposed (Buschmann and Huang, 2010).

Post-acquisition stages (e.g. filtering, amplification, and noise reduction modules), directly integrated in in-ear devices or in post-processing systems, are often required to condition the acquired signals and improve accuracy (Venema et al., 2012; Budidha and Kyriacou, 2014). The energy consumption of optical in-ear device is mainly related to LED driving, analog-to-digital conversion, and microprocessor operations (Vogel et al., 2009). Additional energy costs can be attributed to the presence of wireless communication modules for data transmission to other devices (e.g. smartphones and computers), where applications are available for data visualization and post-processing. Rechargeable long-term batteries in most devices usually guarantee hours of monitoring time.

## Scoping Review Methods

The scoping review and the meta-analysis were conducted following the guidelines of the Preferred Reporting Items for Systematic Review and Meta-Analysis (PRISMA) extension for scoping reviews (PRISMA-ScR) (Tricco et al., 2018).

### Eligibility Criteria

The literature search was performed to identify studies performing and assessing in-ear monitoring of physiological parameters, namely T, HR/PR, and SpO_2_, in the last 10 years. The search strategy design was schematized by the inclusion criteria (Table 1), categorized according to the broad Population—Concept—Context (PCC) mnemonic recommended for scoping reviews (Peters, 2016; Munn et al., 2018). The scoping review was focused on applications in physiological research, sport science, and occupational medicine, including challenging environmental conditions. Thus, only studies on healthy subjects during daily activities were included, while studies considering patients in clinical/rehabilitation care or monitoring of pathological conditions and sleep disorders were excluded. Studies were eligible only if physiological parameters were measured directly from the ear canal, while studies on devices simply worn on the auricle were excluded. Since the reliability of ear canal measurement was a relevant issue, studies were included only if reference/comparative measurements of the same variable were reported. The search was restricted to articles published in English in peer-reviewed journals. No restriction on study design was posed. Abstracts presentations, conference proceedings, and reviews were excluded.

**Table 1:** Inclusion criteria for the scoping review summarized according to the Population-Concept-Context (PCC) mnemonic, recommended for scoping reviews (Peters, 2016; Munn et al., 2018).

Population	• Healthy adults • Any sex
Concept	• In-ear measurement/monitoring of physiological parameters, i.e., - Temperature, - Heart/pulse rate, - Oxygen saturation • Comparison with measurements from other body sites.
Context	• All daily-life, non-clinical settings, with focus on physiological research, sport science, occupational medicine, and challenging environments. • Original peer-reviewed research articles (any study design), published in English in the last 10 years.

### Information Sources, Search Strategy, and Study Selection

A systematic search was performed in Pubmed, Scopus, and ISI Web of Science electronic databases to identify primary references from January 2010 to December 2019. The following search string was used: (“earbud” OR “earpiece” OR “earable” OR “hearable” OR “headphone” OR “ear device” OR “ear sensor” OR “in-ear device” OR “in-ear sensor” OR “ear canal sensor” OR “ear canal device” OR “ear canal” OR “tympanic device” OR “tympanic sensor”) AND (“physiological parameter” OR “physiological monitoring” OR “physiological index” OR “physiological signal” OR “vitals” OR “vital parameter” OR “vital sign” OR “temperature” OR “oxygen saturation” OR “oximetry” OR “oximeter” OR “photoplethysmography” OR “heart rate” OR “heart frequency”). The database search was followed by a review of the citations from eligible studies. Studies were selected based on title and abstract using the online platform Rayyan (Ouzzani et al., 2016). Selected studies were read thoroughly to identify those suitable for inclusion in the scoping review.

### Data Extraction

Two reviewers (MM and AM) independently extracted the demographic and experimental data from the selected studies. When disagreement occurred, they reviewed the papers together to reach joint conclusions. For each study the following relevant information were extracted and summarized: the characteristics of the in-ear device under evaluation and of the reference and/or comparators; the experimental setting/protocol for the evaluation and the investigated study group; the main results of the study with focus on the device performance and/or critical issues.

## Results

### Study Selection

**Figure 3:**
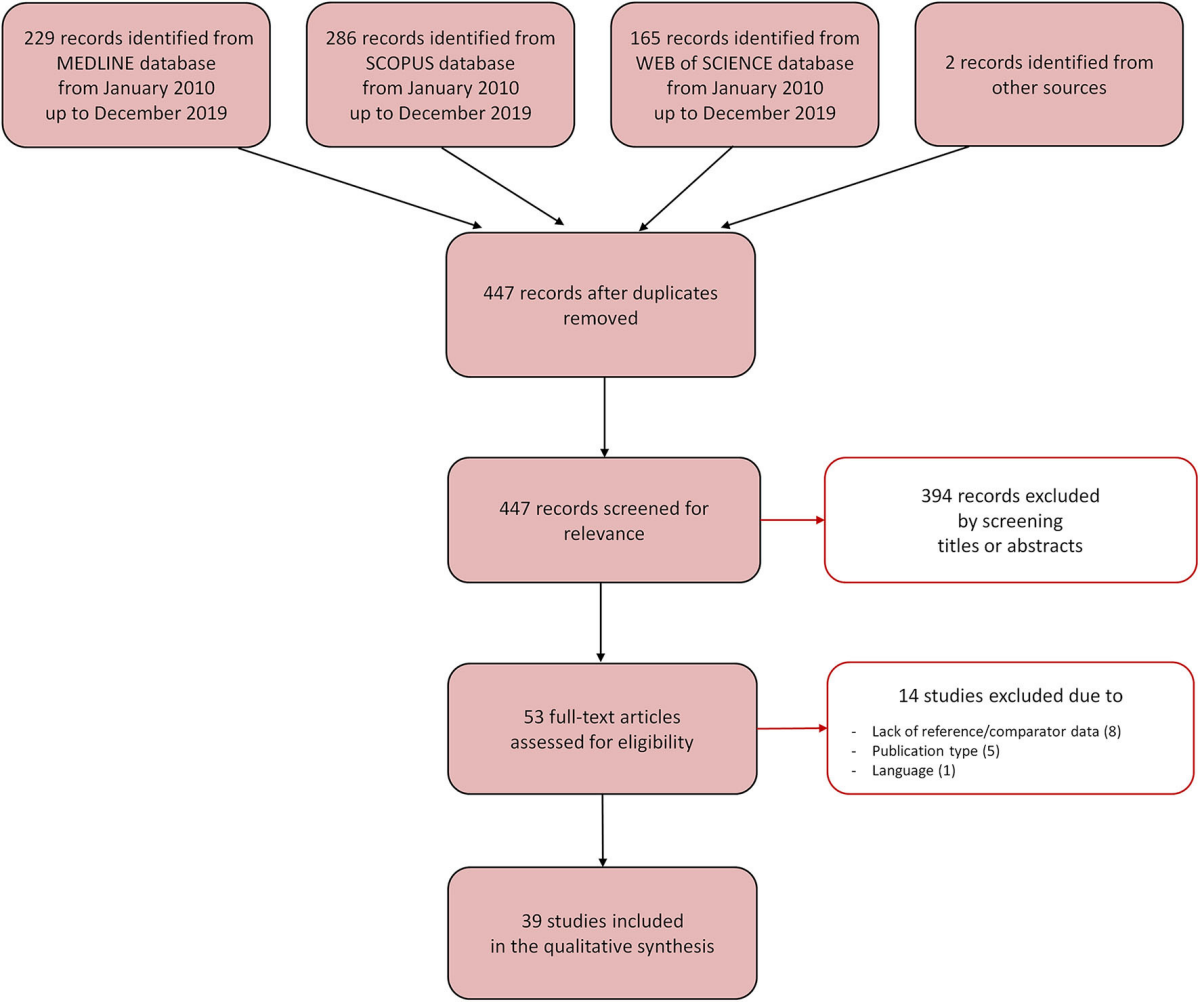
Selection process for the studies included in the scoping review. The Preferred Reporting Items for Systemic Reviews and Meta-Analyses [PRISMA-sc (Tricco et al., 2018)] flow diagram depicts the number of records identified, included and excluded, and the reasons for exclusion, through the different phases of the scoping review.

The database search identified a total of 447 relevant references once the duplicates were removed (Figure 3). A total of 394 references were excluded after reading title and abstract, and 53 were retrieved for further evaluation. Of these, 14 studies were excluded because they did not fulfill the inclusion criteria, mostly due to the absence of reference/comparator measurements (8 studies). Following the selection process, 39 studies were included in the scoping review. Of these, 24 studies performed a measurement of T, 12 studies of HR/PR, and three studies of SpO_2_. The studies are described in the next paragraphs and briefly summarized in Tables 2–4.

**Table 2:** Included studies performing in-ear monitoring of temperature.

**Study**	**Device for in-ear T measurement**	**Comparator/reference**	**Experimental setting and population**	**Main results**
Bagley et al. (2011)	IRTT (Thermoscan ExacTemp IRT 4520, Braun).	Rectal probe.	Rest and exercise in cold environment (7 ± 1°C; 48 ± 5% relative humidity, 150 min) in 25 healthy subjects.	Rectal T significantly exceeded tympanic T (*p* < 0.0005) in all measurements during rest and exercise conditions in cold, with heteroschedasticity between the two measurements (*r*^2^ = 0.181; bias = −1.4°C).
Basak et al. (2013)	IRTT (Genius First Temp M3000A, Tyco Healthcare Group).	Oral probe, forehead non-contact IR thermometer.	Rest condition in 452 healthy subjects at constant room temperature	Strong correlation was observed between tympanic and oral T (*r*^2#^ = 0.75, *P* < 0.01) and tympanic and forehead T (*r*^2#^ = 0.64, *P* < 0.01).
Basset et al. (2011)	Thermocouple thermometer (Mon-a-therm 400 series thermistor, model 90058, Mallinckrodt Medical Inc).	Rectal and esophageal probes, skin thermistors.	T measurement before, during, and after immersion of lower body surface in cold (5 ± 0.1°C) water in a thermoneutral air environment (~21.5 ± 0.5°C) in 12 healthy subjects.	Rectal T significantly dropped from baseline values (−1.0 ± 0.4°C, *P* < 0.05) by the end of the cooling phase, while smaller changes were observed in tympanic (−0.3 ± 0.3°C) and esophageal T (−0.1 ± 0.3°C).
Bhangu and Parmar (2010)	IRTT (Thermoscan IRT 4520, Braun).	Oral probe.	“Tough Guy” outdoor endurance event in 64 athletes (environmental T = 2.8°C; indoor T ~21°C).	Tympanic T was significantly higher than oral T at discharge [35.0°C (95% CI, 34.6–35.3°C) vs. 33.8°C (95% CI, 33.2–34.5), *P* = 0.003]
Chaglla et al. (2018)	Prototype thermometer with graphene-inked MLX90614-DCA IR thermopile and 3D printed ear hook enclosure. Bluetooth module for wireless connectivity to a smartphone application.	Original sensor, Cosinuss One ear thermometer, IRTT (ThermoScan 7AgePrecision IRT652, Braun).	Rest condition in 10 healthy subjects and exercise (total 25 min) in 1 subject, at room temperature (21°C).	The graphene-inked prototype demonstrated higher accuracy and was less influenced by ambient T than commercial sensors. At rest, the mean bias* was −0.36°C for the graphene-inked vs. −0.51°C for the original IRTT. During exercise, the graphene-inked prototype was less affected by airflow and ambient T.
Flouris and Cheung (2010a)	Flexible thermistor thermometer (MA-100, Thermometrics, Edison).	Rectal probe, mean body T (skin heat flow with 6 pen-shaped humidity probes), and mean skin T (12 thermistors)	Rest (1 h) and exercise (to volitional exhaustion on a cycle ergometer at 20 W with step increments of 20 W h^−1^) in environmental chamber at 42°C in 10 healthy males.	Tympanic T linearly increased with rectal T, mean body and mean skin T, albeit with a 100 min delay.
Flouris and Cheung (2010b)	Flexible thermistor (MA-100, Thermometrics, Edison).	Rectal probe and exhaled breath T (chip thermistor inside a valve connected to a mask).	Immersion in water tanks at 42°C/12° C until increase/decrease of rectal T with respect to baseline T, in 15 healthy individuals.	Tympanic T was significantly different (*P* < 0.05) from rectal T and exhaled breath T, albeit it showed good correlation with rectal T (*r*^2#^ ranging from 0.20 to 0.96). Tympanic T displayed moderate delays in repetitive changes of body T in the water tanks.
Fogt et al. (2017)	IRTTs (T1: Welch Allyn Braun Pro 4000, Braun; T2: Covidien Genius2, Covidien).	Gastrointestinal pills, oral and temporal probes.	Graded exercise until voluntary exhaustion in an environmental chamber (35.5 ± 0.6°C, 53.9 ± 5.8% relative humidity) 14 young, active and healthy adults.	No differences were observed between mean T1 and pill T (LoA = ±1.90°C), while mean T2 was significantly higher (*P* = 0.008) than pill T (LoA = ±2.15°C).
Gagnon et al. (2010)	Thermocouple probe thermometer (Mon-a-therm, model 503-0021, Covidien-Nellcor).	Rectal and esophageal probes.	Exercise in the heat (42°C, 30% relative humidity) till hyperthermia in 24 healthy subjects. Subsequent immersion in water (26°) until rectal T reached 37.5°C (12 subjects) or recovery in a temperate environment (30°C, 30% relative humidity) for 60 min (12 subjects).	At the end of the exercise session in the heat, tympanic T was significantly lower than esophageal T (*P* < 0.001), but was not significantly different from rectal T. In the subsequent cold immersion or recovery stage, tympanic T was significantly lower than rectal T (*P* < 0.001), but was not significantly different from esophageal T.
Harmanci et al. (2018)	IRTT (Thermoscan ExacTemp IRT 4520; Braun).	Gastrointestinal pills.	Rest and two treadmill exercise sessions in a climate- controlled exercise laboratory (45–50% relative humidity and 22–25°C room T) in 15 healthy female futsal players.	No significant difference was observed between gastrointestinal and tympanic T at rest (*P* > 0.05). Tympanic T was significantly lower than gastrointestinal T after 10, 20, and 30 min of exercise (*P* < 0.05).
Kallmünzer et al. (2011)	IRTT (Genius 2, Tyco Healthcare Group).	Rectal probe.	Gel-based neck cooling for 190 min in 10 healthy subjects.	After neck cooling, a significant drop from baseline (−1.7°C, *P* = 0.001) was observed in tympanic T vs. a smaller decrease (−0.65°C, *P* = 0.019) in rectal T, concurrent with a decrease in HR of 15 bpm.
Keene et al. (2015)	IRTT (ThermoScan IRT 4520, Braun).	Gastrointestinal probe.	Two 20 min work in heat chamber set at 100°C in 37 professional firefighters.	Tympanic T underestimated gastrointestinal T by 1.3 ± 0.5°C before entering the chamber, and by 1.0 ± 0.8°C following exercise.
Lee et al. (2011)	IRTT (CE Thermo, Nipro Corporation) equipped with a silicon mold to fit into the ear. Outer ear tightly sealed using surgical tape. Wireless transmitter and receiver for telemetric system.	Rectal probe.	12 conditions: 2 activities × 3 clothing levels × 2 air T (25°C and 32°C with 50% relative humidity). WBGT: 19.4 and 25.4°C for 25 and 32°C, respectively, in 8 healthy males.	Although changes in tympanic T were significantly (*P* < 0.05) greater than those in rectal T for different clothing levels at rest, an overall agreement between the two T measurements was found. During exercise, tympanic T was lower than rectal T for control and HDPE conditions, but finally reached to/exceeded it for PVC conditions at both 25 and 32°C.
Morán-Navarro et al. (2019)	IRTTs (T1: Braun Thermoscan IRT6520, Braun; T2: JPD-FR100, Etekcity).	Oral and skin probes, ingestible sensor.	Outdoor 60 min submaximal exercise in the heat (40.1 ± 0.5°C, 39.5 ± 3.4% relative humidity) on cycle ergometer in 12 well-trained athletes.	T1 registered temperatures similar to the ingestible core T sensor at rest (bias* = 0.1°C; P = NS) and during exercise in the heat with (bias* = −0.1°C; *P* = NS) and without wind (bias* < −0.1°C; *P* = NS), but it registered colder T during recovery (bias* = −0.7°C; *P* < 0.001). T2 registered colder T values at rest (bias* = −1.4°C; *P* < 0.001) and during recovery (bias* = −1.1°C; *P* < 0.00), but warmer T during exercise in the heat without wind (bias* = 0.9°C; *P* < 0.001).
Muth et al. (2010)	IRTT (First Temp Genius, Sherwood Medical).	Sublingual probe.	Swimming (45 min water temperature 28°C) with immersed ears or not, in 25 healthy subjects.	At baseline, sublingual T was significantly lower than tympanic T in immersed (*P* < 0.001) and control (*P* = 0.002) groups. In the head-immersed group, sublingual T was significantly (*P* < 0.001) higher than tympanic T. In the head-not-immersed group, sublingual T was significantly lower than tympanic T (*P* = 0.002).
Nagano et al. (2010)	Tympanic thermocouple probe in earplug (3M 1110, 3M Health Care).	Esophageal, skin, and rectal probes.	Work-rest cycle simulation in warm environment (climatic chamber) in 6 healthy subjects.	Tympanic T successfully tracked rectal T during the work-rest-work simulated cycles, with slight T underestimation (bias* = −0.45 ± 0.08, −0.36 ± 0.11, and −0.3 ± 0.12 at ambient T of 25, 30, and 35°C, respectively).
Nakada et al. (2017)	Customized IRTT embedded into an earplug, measuring T at three different locations in the external auditory canal.	Esophageal and rectal probes.	Exercise (at 80 W for 45 min) in a climatic chamber (24, 32, and 40°C, at 50% relative humidity) in 11 healthy male volunteers.	Tympanic T showed progressively lower values from proximal to distal positions from the tympanic membrane (mean T values decreased from 36.86 ± 0.78°C to 36.40 ± 1.24°C to 35.12 ± 2.82°C at the three locations). Tympanic T was markedly affected by ambient T and radiation. The combination of T at proximal and distal location from the tympanic membrane can help to better estimate esophageal T.
Ota et al. (2017)	Customized 3-D printed IRTT. Bluetooth module for wireless connectivity to a smartphone application.	Skin IR thermometer, commercial IRTT.	Exercise in environmental chamber at T ranging from 6 to 40°C in 1 healthy subject.	IRTT not affected by external perturbations.
Pryor et al. (2012)	IRTT (T1: Thermoscan, model IRT 3520, Braun) and thermocouple probes (T2s: Mon-a-therm Tympanic T probe, thermistor YSI 400 Series, and Mon-a-therm Model 4070, Mallinckrodt Medical Inc).	Gastrointestinal pills, temporal artery probe, skin forehead probe, skin thermistors.	Treadmill walking protocol (<50 min) with thermal protective clothing in warm room (38.2 ± 0.9°C, 22.2 ± 3.9% relative humidity) in 50 firefighters. Subsequent forearm immersion in refrigerated water (5°C) in 25 subjects.	Both IRTT and thermocouple probes greatly underestimated deep gastric T (T1: bias = −1.31°C; LoA = 2.75°C; T2: bias = −3.28°C, LoA = 5.00°C).
Skaiaa et al. (2015)	Tympanic thermistor-based probe (Métraux®).	Rectal probe.	Exposition to four simulated in-field conditions with local cooling of head/ears (ambient air/wind without insulation, ambient air/wind with insulation, snow in ear canal with insulation, cold water in ear canal with insulation) in 13 healthy subjects.	At baseline, tympanic T significantly lower (*P* < 0.001) than rectal T (bias = 0.8°C). After 5 and 10 min of cold exposure, tympanic T was significantly lower (*P* < 0.006) than baseline T, and its significantly underestimated rectal T (bias* = −1.5/−3.2°C at 5 min and = −1.2/−2.0°C at 10 min). After surface cooling of head and neck, tympanic T did not accurately reflect rectal T within the first 10 min of measurement, with risk of over-triage.
Strapazzon et al. (2015)	Tympanic thermistor-based probe (M1024233, GE Healthcare).	Esophageal probe.	Exposition to change in ambient T from normal (23.2 ± 0.4°C) to very low (−18.7 ± 1.0°C), with and without insulation by ear protectors, in 31 healthy subjects.	Tympanic T was influenced by ambient T with increasing deviation from esophageal T at low ambient T (*P* = 0.007 and *P* < 0.001 without and with ear insulation, respectively). Ear insulation reduced T bias by 82% (from 7.2 to 1.3°C) at low T.
Suzuki et al. (2010)	IRTT (EM-30CPLB, Terumo Corporation).	Axillary thermometer, facial thermography.	Rest in cold (12.6°C, 31% relative humidity) and warm (20°C, 36% relative humidity) environments in 50 healthy subjects.	Tympanic T was significantly lower than axillary T in cold environment, and higher than facial T in cold and warm environments (*P* < 0.01). The ROC curve for tympanic T with axillary T as reference had AUC of 0.62 and 0.74 in cold and warm condition, respectively.
Teunissen et al. (2011)	Tympanic thermistor (P-8432, ICBT) mounted bilaterally inside a customized silicon mold, with or without protection from the environment with a cotton patch covering the complete auricle.	Esophageal and rectal probes.	Rest at 21, 10, and 30°C (50% humidity), followed by cyclo-ergometer exercise and recovery at 30°C in 7 subjects. Protocol repetition with and without face-wind during the rest period at 30°C. Extra auricle insulation at one side.	Ambient temperature affected significantly tympanic T, while rectal and esophageal T remained stable. Insulating the auricle mitigated but did not abolish the effect, nor protected from the wind. Acceptable agreement with rectal T was observed during recovery from exercise without wind (bias = −0.66 ± 0.21°C covered vs. −1.20 ± 0.15°C uncovered), while differences increased significantly with wind (bias = −1.73 ± 0.11°C covered vs. −2.49 ± 0.04°C uncovered).
Yamakoshi et al. (2013)	Microminiature IR thermo-pile sensor (10TP583T, Ishizuka Electronics Corp) and thermistor (SXK-67 & SZL-64, Technol Seven Co), molded with a micro-speaker in soft silicon material. Connectable with race face helmet.	Gastrointestinal pills.	Simulated racing environment and after a rest period of 10 min underwent a bathing period of 30 min, with the water preset to 42°C and then a 40 min period, with body T gradually reduced by natural cooling in the laboratory in 10 healthy volunteers. Real racing conditions in 2 professional drivers.	Good agreement between the tympanic IR thermopile sensor and both the thermistor (*r*^2#^ = 0.97, *P* < 0.001; bias* = −0.01°C) and the gastrointestinal T (*r*^2#^ = 0.86, *P* < 0.001; bias* = −0.27°C), respectively. The system functioned well during real competitive racing conditions.

*Agreement between measurements is given as bias [mean, mean ± standard deviation, or median (95% limits of agreement, LoA)], calculated as (tympanic T-reference/comparator T), area under the curve (AUC) of the receiver-operating characteristic curve (ROC), and coefficient of determination (r^2^), as available*.

Bpm, beats per minute; HDPE, high-density polyethylene coverall; HR, heart rate; IRTT, infrared tympanic thermometer; min, minutes; NIRS, near infrared-red spectroscopy; NS, non-significant; P, p-value; PVC, polyvinyl chloride coverall; T, temperature; WBGT, wet bulb globe temperature. ^#^Calculated from r values in the studies; *bias sign reversed with respect to the original article to indicate (tympanic T—reference/comparator T).

**Table 3:** Included studies performing in-ear monitoring of heart/pulse rate.

**Study**	**Device for in-ear HR measurement**	**Comparator/Reference**	**Experimental setting and population**	**Main results**
Boudreaux et al. (2018)	PPG-based sensors (Bose SoundSport Pulse (BSP) Headphones, Bose Corporation). Bluetooth module for wireless connectivity to a smartphone application.	Six wrist-worn and one chest strap device; ECG.	Separate trials of graded cycling and three sets of four resistance exercises at a 10-repetition-maximum load in 50 healthy subjects.	The chest-strap and BSP devices displayed overall best performance (cycling: *r*^2#^ = 0.62; MAPE = 6.87%; resistance exercise: *r*^2#^ = 0.69; MAPE = 6.31%). BSP device displayed best performance during resistance exercise: (*r*^2#^ = 0.74; MAPE = 6.24%). All devices underestimated HR at increasing exercise intensity.
Bunn et al. (2019)	PPG-based sensor (Jabra Pulse, Jabra). Bluetooth module for wireless connectivity to a smartphone application.	Wrist PR monitor; chest strap.	Exercise (30 min treadmill, 25 min high-intensity exercise, 40 min continuous outdoor activity) in 22 active subjects.	Jabra Pulse device displayed good agreement with the chest strap during the treadmill (*r*^2^ = 0.94; bias = 0.8 bpm) and outdoor sessions (*r*^2^ = 0.95; bias = 0.8 bpm), with slightly reduced performance during high-intensity exercise (*r*^2^ = 0.86; bias = −3.6 bpm). It outperformed the wristwatch in all exercise sessions and especially during the high-intensity session.
de Graaf et al. (2019)	Prototype IR thermopile sensor.	ECG.	Rest condition in 5 healthy subjects.	The thermopile sensor displayed poor agreement with the ECG (bias within ±10.5 bpm in 70% of measurements).
Goverdovsky et al. (2017)	Prototype mechanical PPG-based sensor.	ECG from the hands. PPG from the finger.	Rest condition in 3 healthy subjects.	The mechanical PPG sensor displayed high agreement with the ECG (*r*^2#^ = 0.98).
Higgins et al. (2018)	PPG-based sensor (BioConnected, BioConnected). Bluetooth module for wireless connectivity to a smartphone application.	Chest strap.	Exercise (Bruce protocol GTX: maximal graded exercise testing on a treadmill) in 15 healthy subjects.	BioConnected device displayed high agreement with the chest strap (*r*^2#^ = 0.94). As exercise intensity increased the device showed occasional displacement problems.
Leboeuf et al. (2014)	Prototype optomechanic PPG-based sensor plus accelerometer (Performtek), with “medallion” housing microcontroller and Bluetooth transmission module.	Chest strap.	Exercise (rest, sitting, treadmill exercise at graded intensity) in 14 (training) + 9 (test) healthy subjects.	The prototype device displayed high agreement with the ECG throughout the full range of activities (*r*^2^ = 0.98; bias = −0.2 ± 4.4%)
Park et al. (2015)	Prototype piezoelectric sensor. Cross-shaped hardware digital circuit housing micro-controller plus wireless communication module and coin-cell battery on the apparatus body.	ECG.	Rest sitting condition in 58 healthy subjects.	The prototype device showed a high level of agreement with the ECG at rest in stable conditions (*r*^2^ = 0.91). Performance was negatively affected by motion.
Passler et al. (2019)	PPG-based sensors (Cosinuss One, Cosinnus; Dash Pro, Bragi). Bluetooth module for connectivity to a smartphone application.	ECG.	Exercise (10 min rest + 20 min subject-specific stress test on a cyclo-ergometer) in 20 healthy subjects.	Cosinnus One and Dash Pro devices displayed good agreement with the ECG at HR <90 bpm (MAPE = 2.5 and 3.2%) and at HR >100 bpm (1.3 and 1.4%). Both devices slightly underestimated HR values, and were sensitive to motion artifacts.
Poh et al. (2012)	Prototype dual-ear reflective photosensor. Serial and Bluetooth connection to laptop/smartphone application.	ECG.	Basal standing, exercise 1 (2 min sitting, 5 min cycling, 2 min sitting on recumbent bike), exercise 2 (2 min standing, 5 min walking, 2 min standing on treadmill), music playing in 31 healthy subjects.	The prototype device displayed good agreement with the ECG during basal standing (bias = −0.07 bpm (−5.09; 4.95), exercise 1 [bias = 0.67 bpm (−3.92; 5.27)], and exercise 2 [bias = 0.51 bpm (−9.89; 10.9)].
Tomita et al. (2018)	Prototype dual-ear PPG-based sensor plus accelerometer. Wireless connectivity to a signal processing application in device.	ECG.	Exercise (3 min mouth opening and closing, 3 min head-shake, 3 min walking, 17 min running) in 6 healthy subjects*	The dual sensor device in combination with asynchronous noise removal displayed good agreement with the ECG (bias within ±5 bpm in 95% of measurements).
Vogel et al. (2009)	Prototype PPG-based sensor (IN-MONIT), connected with a body box containing signal acquisition and communication circuitry.	Finger clip sensor.	Rest condition in 1 healthy subject.	The prototype device combining IR PPG signal acquisition with frequency-based algorithms displayed best agreement with finger clip sensor (*r*^2#^ = 0.85, *P* < 0.001; bias = −0.59 ± 1.33 bpm).
von Rosenberg et al. (2017)	Prototype electrode plus microphone sensor.	ECG. Head-ECG	Rest condition in 5 healthy subjects.	The prototype device provided an ear-ECG signal with identifiable waves, although signal amplitude was 1/50 of Lead 1 ECG. The ear-ECG extracted cardiac cycles correlated well with Lead1 cardiac cycles, with *r*^2#^ = 0.92 and 0.81, respectively, when using Lead 1 or microphone signals to guide R-wave identification.

*Agreement between measurements is given as bias [mean, mean ± standard deviation, or median (95% limits of agreement)], calculated as (tympanic HR-reference/comparator HR), coefficient of determination (r^2^), and/or mean absolute percentage error (MAPE), as available*.

Bpm, beats per minute; ECG, electrocardiogram; HR, heart rate; IR, infrared; min, minutes; P, p-value; PPG, photopletysmographic signal; ^*^Detailed protocol from (Tomita, 2016); ^#^Calculated from r values in the studies.

**Table 4:** Included studies performing in-ear monitoring of oxygen peripheral saturation.

**Study**	**Device for in-ear SpO_**2**_ measurement**	**Comparator/Reference**	**Experimental setting and population**	**Main results**
Budidha and Kyriacou (2014)	Prototype reflection-based, dual wavelength sensor.	Finger sensor.	Cold immersion (1°C ice bath) test in 15 healthy volunteers.	During ice water immersion, no significant change was observed in red and IR ear PPG amplitudes (+2.5 and −1.2%, respectively) vs. a significant drop for red and IR PPG from right (52.7 and 58.3%) and left index fingers (47.5 and 46.8%).
Budidha and Kyriacou (2018)	Prototype reflection-based, dual wavelength sensor.	Finger sensor.	Cold environmental condition (10°C) in 15 healthy volunteers.	During cold exposure, only slight reduction of red and IR ear PPG normalized amplitudes (0.2 and 13%, respectively) was observed vs. a drop in finger PPG amplitude (>80%).
Venema et al. (2012)	Prototype reflection-based, dual wavelength sensor.	Finger and forehead sensors; blood gas analysis.	Hypoxic condition (blood desaturated in five steps from ~100 to 70–77%) in 10 healthy volunteers.	Under hypoxic conditions, in-ear SpO_2_ measures obtained by single-point calibration displayed good agreement with blood gas analysis values (*r^2^* = 0.96; MSE = 3.15).

*Agreement between measurements is given as coefficient of determination (r^2^), and/or mean squared error (MSE), when available*.

*IR, infrared; PPG, photoplethysmographic signal; SpO_2_, oxygen peripheral saturation*.

### Measurement of Temperature

#### Introduction

The monitoring of T is an essential component of physiological research and sport science, and a fundamental diagnostic parameter in emergency and occupational medicine for guiding treatment and triage decisions (Pasquier et al., 2019; Strapazzon et al., 2019). In sport science, the measurement of core T is critical when evaluating research questions related to heat production, such as heat acclimatization and body cooling, exercise intensity, and effect of different environmental conditions on physiological state and performance. Monitoring of core T can be used to guarantee intensity and safety during intense heat exercise and heat tolerance tests, and to prevent exertional heat stroke (American College of Sports Medicine et al., 2007) or unexpected cooling (Procter et al., 2018). Indeed, a decrease in core T *per se* may significantly impair physiological functions even in a healthy subject and should be timely detected for proper triage (Danzl and Pozos, 1994; Paal et al., 2018). On the other hand, core T is considered a pivotal parameter also in occupational medicine, for instance to identify subjects at risk of heat stress disorders (Nagano et al., 2010).

Although invasive measurements, such as those performed in the pulmonary artery or lower third of the esophagus, remain the gold standard for assessment of core T, these sites are not practical in many situations, including the described settings (Strapazzon et al., 2014). The ear canal was proposed as a promising alternative site to measure core T, thanks to the functional relation between the TM and the hypothalamic central thermoreceptors via shared blood supply (McCarthy and Heusch, 2006), the non-invasivity, the respect of basic hygiene standards, the relative independence from external environmental conditions, the wearability, and the fast monitoring response (Gunga et al., 2009; Strapazzon et al., 2014).

#### Results and Discussion

Twenty-four studies, summarized in Table 2, analyzed the suitability of the ear canal as measuring site for core T under different physiological and environmental conditions. The works differed in terms of sensors (IR or thermistor-based devices) and device technology (market or prototype devices), the core T reference/comparator (e.g., esophageal, rectal, or gastrointestinal), as well as for the protocols and scenarios within which the measurements were performed. As well, differences in populations (i.e., healthy volunteers or athletes) and demographic variables among studies were present, which may have introduced further variability in T measurements (Heusch et al., 2006; McCarthy and Heusch, 2006).

Most of the studies (sixteen) performed in-ear T monitoring by IR thermometers. IR measurements were shown to display high correlation with oral and forehead measurements in resting individuals (*p* < 0.01) (Basak et al., 2013). More variable results were obtained in the studies analyzing the effect of exercise in different environmental conditions (Gagnon et al., 2010; Bagley et al., 2011; Fogt et al., 2017; Harmanci et al., 2018; Morán-Navarro et al., 2019), where microclimate changes in the ear canal and changes in blood flow to the skin are known to impact measurements (Patel et al., 1996; Jensen et al., 2000; Casa et al., 2005; Kistemaker et al., 2006). Of note, tympanic measurements of T were not always compared to the “gold standard” site for core T (esophageal) (Strapazzon et al., 2014). Tympanic probes were shown to display different performance—with respect to other T monitoring sites—when compared to ingestible sensors during rest, exercise, and recovery (also under wind conditions) with a bias ranging from −0.7 to 1.4°C (*p* < 0.001) (Morán-Navarro et al., 2019). Tympanic devices reflected core T measured by gastrointestinal pills in 14 healthy subjects exercising in a hot, humid environment, although different IR tympanic thermometers displayed diverse response (limits of agreement varying from ± 1.90 to ± 2.15°C), highlighting the need for appropriate selection and validation of single devices (Fogt et al., 2017). Harmanci et al. demonstrated that tympanic T tracked lower values than ingestible sensors during the progress of exercise (*p* < 0.05) (Harmanci et al., 2018). Bagley et al. observed that tympanic T was significantly lower than rectal T during both rest and exercise under cold experimental conditions (bias = −1.4°C, *p* < 0.0005) (Bagley et al., 2011). Gagnon and co-workers found that tympanic T increased at a rate similar to rectal T, but was consistently lower than esophageal T (*p* < 0.01) in healthy subjects exercising in a hot temperature-controlled chamber (Gagnon et al., 2010). When immersing subjects in cold water or during recovery, tympanic T remained significantly lower than rectal T, suggesting an effect of physiologic differences in regional blood flow during and after exertional heat stress and of the buffering influence of rectal dense tissue mass around the probe (Gagnon et al., 2010). Overall, these results should be interpreted with caution since rectal and gastrointestinal sites have different behaviors and known limitations in dynamic conditions that may limit their validity as T reference/comparator with respect to the esophageal site (Lee et al., 2000; Lim et al., 2008; Wilkinson et al., 2008; Strapazzon et al., 2014).

Effects on tympanic T measurements of local temperature changes in areas proximal or distant from the ear were further investigated in four studies using either IR (Kallmünzer et al., 2011) and thermistor-based thermometers (Flouris and Cheung, 2010a,b; Suzuki et al., 2010; Basset et al., 2011). Local cooling of the head and neck was shown to modify tympanic T measurement to a larger extent than rectal T (−1.7 and −0.65°C from baseline, respectively, *p* < 0.05) (Kallmünzer et al., 2011). On the other hand, Basset et al. showed that tympanic and esophageal T were almost unaffected (−0.3 ± 0.3°C and −0.1 ± 0.3°C from baseline, respectively) by cooling of the lower body surface (Basset et al., 2011), while rectal T underwent a significant drop (−1.0 ± 0.4°C from baseline, *p* < 0.05). These results were further expanded in two studies by Flouris and Cheung, who investigated tympanic T changes in healthy subjects performing exercise or entering hot and cold water tanks (Flouris and Cheung, 2010a,b). Although delayed with respect to rectal, skin, and exhaled breath T, and different in absolute values (*p* < 0.05), tympanic T measurements displayed a significant agreement and correlation with other monitoring sites (coefficient of determination *r*^2^ ranging from 0.20 to 0.96 with rectal T).

Five studies analyzed the effects of cold environments on tympanic T measurements, in simulated conditions (Teunissen et al., 2011; Skaiaa et al., 2015; Strapazzon et al., 2015) and sport/competition settings (Bhangu and Parmar, 2010; Muth et al., 2010), to investigate the device suitability for hypothermia prevention. Tympanic T, measured by a commercial tympanic thermistor sensor under different environmental conditions (i.e., ambient air with or without local/wind insulation; snow or icy water in ear canal) displayed significantly (*p* < 0.006) lower values than rectal T (bias ranging from −1.5/−3.2°C at 5 min to −1.2/−2.0°C at 10 min) (Skaiaa et al., 2015). Similar results were obtained by Strapazzon et al. comparing a tympanic probe to an esophageal one. However, ear insulation was able to reduce bias by 52% (from 2.9 to 1.5°C) in the ambient setting and by 82% (7.2–1.3°C) in the low T setting (Strapazzon et al., 2015). An inter-individual variability was observed in the degree of deviation of tympanic from esophageal T (Strapazzon et al., 2015), which was consistent with variability in physiological factors (vascularization, conductivity, and tissue perfusion) and anatomical differences (length, width, shape) affecting probe placement. The impact of cold environmental conditions on tympanic T and the mitigating effect of insulating the ear thermistor from environmental changes were confirmed during a rest, exercise, and recovery protocol (Teunissen et al., 2011). Of note, wind had an immediate cooling effect on tympanic T, which was not sufficiently counteracted by insulation (bias = −1.73 ± 0.11°C and −2.49 ± 0.04°C in covered and uncovered ear condition, respectively) (Teunissen et al., 2011). The negative impact of extreme environmental conditions on tympanic measurements was observed also for IR probes in cold environments, such as swimming in cold water (Muth et al., 2010) and outdoor endurance events (Bhangu and Parmar, 2010). In relation to swimming, it was suggested that the water in the ear canal could significantly influence T measurements, either by creating a cool “buffer zone” between the probe and the TM and/or by cooling down the ear canal wall and the TM (Muth et al., 2010).

Given the limitations of IR thermometers, three technical studies proposed technological improvements to address the effect of anatomical and environmental factors on T measurements (Yamakoshi et al., 2010; Ota et al., 2017; Chaglla et al., 2018). To reduce the impact of individual ear morphology and environmental conditions, Chaglla et al. (2018) proposed a graphene-inked sensor, obtained from a commercial IR thermopile and fixed by a 3D-printed hook-type enclosure. The sensing device was developed to continuously measure T from the TM and to display it on a smartphone. It included a microcontroller processing unit and a Bluetooth module for wireless connectivity to a smartphone application and was powered with a rechargeable lithium-polymer battery (3.7 V) providing continuous operation for at least 4 h. The sensor reduced the influence of ambient T (mean bias = −0.36°C), with respect to a reference commercial sensor (mean bias = −0.51°C). Similar to these results, Ota et al. showed that a wearable IR sensor, combined with a hearing aid and inserted in a 3D-printed device, could provide a more reliable T estimation than a skin sensor (Ota et al., 2017). Together with the sensor module the device included a Bluetooth transceiver (powered by a 3.7 V lithium battery) for wireless data transmission to a mobile application interface displaying T values in real time. This approach was further corroborated by results from Yamakoshi et al., who reported good correlation of T values measured by an in-ear customized molded IR sensor with values measured by a thermistor-based tympanic sensor (*r*^2^ = 0.97, *p* < 0.01, bias = −0.01°C) and by gastrointestinal pills (*r*^2^ = 0.86, *p* < 0.001, bias = −0.27°C) in subjects under hyperthermic conditions. The earpiece device, connected to a full-face helmet, sensor amplifier, and signal processor, was also preliminarily tested in-field on professional drivers during real racing conditions and displayed good technical functioning, although no comparison with core T were performed in this setting (Yamakoshi et al., 2013).

Five studies analyzed the potential of in-ear T measurements for occupational medicine for prevention of heat illness (Nagano et al., 2010; Lee et al., 2011; Pryor et al., 2012; Keene et al., 2015; Nakada et al., 2017). The studies reported controversial results. Nagano et al. showed that tympanic T, measured by a thermocouple probe with tight sealing in the ear canal, closely tracked both esophageal and rectal T with slight underestimation during simulated rest-work cycles at increasing environmental T (Nagano et al., 2010). IR tympanic probes underestimated core T obtained by gastrointestinal pills in firefighters performing work in heat chambers (bias ranging from 1.3 ± 0.5°C to 1.0 ± 0.8°C when entering the chamber and following exercise) (Keene et al., 2015) and during exercise with thermal protective clothing in a warm room (Pryor et al., 2012). By using a telemetry system consisting of an ear probe with IR sensor technology, a transmitter, and a wireless data receiver, Lee and co-workers showed an overall high level of agreement between tympanic and rectal T, both at rest and during exercise conditions, in healthy volunteers, although bias varied with clothing levels. Tympanic T was lower than rectal T in control conditions and when wearing a high-density polyethylene coverall, while it reached and exceeded rectal T for a polyvinyl chloride coverall at environmental T of 25 and 32°C (Lee et al., 2011). Nevertheless, given the slow response of rectal measurements, comparisons of tympanic and rectal T away from steady-state conditions should be interpreted with caution (Lee et al., 2000; Lim et al., 2008; Strapazzon et al., 2014). Interestingly, by using a customized IR device, equipped with a silicon mold to fit into the ear and capable of measuring T at three different locations from the TM, Nakada et al. demonstrated that inner and outer T measurements along the ear canal could accurately estimate esophageal T under different physiological and environmental conditions simulating elevated ambient T (Nakada et al., 2017).

### Measurement of Heart/Pulse Rate

#### Introduction

The measure of HR/PR, eventually complemented by heart rate variability parameters, represents an inexpensive, non-invasive, and time-efficient way to monitor cardiovascular performance and autonomic nervous system status (Buchheit, 2014). In sport science, measures of resting, exercise, and recovery HR are used as surrogate markers of fatigue, fitness, and endurance performance status, with implications for optimization of training loads and enhancement of performance (Buchheit, 2014; Schneider et al., 2018). Thanks to the wide diffusion of PPG technology in wearable sensors, PR has become a readily available parameter and real-world PR values, acquired during daily-life activities, are gaining increasing importance (Poh and Kittler, 2012; Bunn et al., 2019). However, acquisition of PPG signals and accuracy of PR values during physical activity and exercise modes in indoor or outdoor settings may be compromised by motion artifacts, noise, and reduced signal quality (Poh et al., 2010; Tomita, 2016). The ear canal bears potential for HR/PR monitoring during exercise, since the head is less affected by motion artifacts, orthostatic pressure modulations, and low perfusion problems than other extremities.

#### Results and Discussion

Twelve studies reporting data on earbud devices for HR/PR monitoring are summarized in Table 3. The majority (eight) of the studies used PPG-based sensors for monitoring PR, one study used electrodes and a microphone embedded in a earbud for measuring an ear-ECG (von Rosenberg et al., 2017), and three studies proposed alternative techniques, such as IR thermography (de Graaf et al., 2019), mechanical photoplethysmography (Goverdovsky et al., 2017), and pressure measurement by piezoelectric sensors (Park et al., 2015). Although non-optical methods may have the advantage of no light source and reduced power consumption, the performed studies suggested the feasibility of HR/PR measurements only under basic resting conditions in small groups of subjects. Sensitivity to user's motion may impair performance under exercise conditions, requiring further optimization of the electronics and sensor placements within the earpiece, and noise reduction in the readout circuitry. On the other hand, methods allowing to acquire an ECG, instead of a pulsatile signal, suggested the possibility to trace cardiac activity beyond HR estimation. After demonstrating in a simulation model the possibility to trace ECG from ear or head locations, von Rosenberg et al. showed in resting subjects that cardiac cycles extracted from ear-ECG correlated well with those extracted from Lead 1 ECG. Although better performance was obtained using the Lead 1 ECG to guide R-wave identification (*r*^2^ = 0.92), good performance could be achieved also for the stand-alone device, where the microphone signal was used to guide wave identification (*r*^2^ = 0.81) (von Rosenberg et al., 2017).

A more extensive validation of in-ear devices, including tests during exercise sessions at different intensity levels, was performed in the studies using PPG-based sensors. Four studies evaluated the performance of commercial in-ear devices (Boudreaux et al., 2018; Higgins et al., 2018; Bunn et al., 2019; Passler et al., 2019). Bunn et al. (2019) compared the performance of a commercial earbud system and a wrist-watch for PR monitoring with that of a chest strap used as benchmark. The earbud device, transmitting information via Bluetooth to a smartphone, demonstrated consistent performance throughout three exercise protocols (bias ranging from 0.8 to −3.6 bpm during treadmill/outdoor sessions and high-intensity session, respectively), with slightly reduced agreement with the benchmark during high-intensity exercise where it slightly underestimated PR. The earbud displayed higher performance than the wrist-watch in all exercise sessions but especially in the high-intensity session, where the wrist-watch exhibited poor performance due to substantial arm motion. High agreement between a commercial wireless exercise earpiece (connected via Bluetooth with a smartphone) and a reference chest strap (*r*^2^ = 0.94) was reported by Higgins et al. during maximal graded exercise on a treadmill (Higgins et al., 2018). However, the authors pointed out occasional displacement problems of both devices when exercise intensity increased. Passler et al. compared the performance of two commercial in-ear PR trackers with a reference ECG at rest and during subject-specific stress tests on a cyclo-ergometer (Passler et al., 2019). Both devices displayed accurate PR measurements in either condition (mean absolute percentage errors ranging from 2.5 to 3.2% for PR <90 bpm and from 1.3 to 1.4 for PR >100 bpm), despite a tendency to PR underestimation and exposure to motion artifacts. An extended comparison of eight commercial PR monitoring systems, including an in-ear device which transmitted real-time PR data via Bluetooth to a smartphone application, was performed by Boudreaux et al. during trials of graded cycling and standard resistance exercise at maximal load (Boudreaux et al., 2018). The devices displayed different performance under different exercise conditions. Only the in-ear device and a chest-worn device displayed limited bias with respect to ECG data in both cycling and resistance exercise sessions, the in-ear device displaying the best performance during the resistance exercise (mean absolute percentage error of 6.24%). In all devices a greater underestimation of HR was observed as exercise intensity increased.

Two additional studies implemented and tested prototype devices for PR monitoring (Poh and Kittler, 2012; Leboeuf et al., 2014). Poh and Kittler (2012) developed the “Heartphone system,” embedding a reflective IR photosensor in a fully-integrated unobtrusive wireless headset, powered by a rechargeable lithium polymer battery. The system architecture included a processing control unit with active filters to reduce electrical noise and motion artifacts, an analog-to-digital converter, a PPG peak detection algorithm, and a communication system (either serial and wireless) to send PR values to a custom application on mobile devices or laptops. The device showed robust PR measurements under conditions of moderate motion, with high agreement with ECG-derived HR measurements when users were standing, cycling, or walking (bias ranging from −0.07 to 0.67 bpm), but lower performance was expected under vigorous exercise. Leboeuf et al. (2014) tested an earbud PPG sensor including an accelerometer. The earbud was designed to be pluggable via a detachable connector to a wireless “medallion,” which housed a microprocessor and a Bluetooth chipset, and granted several hours of measurement time. The device, tested during a treadmill exercise protocol including rest to peak performance steps, showed accurate PR monitoring throughout all activity levels with high correlation (*r*^2^ = 0.98) and low bias (−0.2 ± 4.4%) when compared with the benchmark ECG device. High performance in PR estimation also during extreme physical activity was obtained thanks to the capability of characterizing motion noise through the accelerometer and of attenuating motion artifacts from the optical signal in real-time. To provide a thorough characterization of the physiological state during exercise, the authors combined PR and contextual accelerometry values in a statistical model to estimate total energy expenditure and aerobic capacity. Total energy expenditure could be reliably estimated, while lower accuracy was observed for aerobic capacity.

Technical aspects related to PPG signal filtering and PR estimation from in-ear devices were addressed in two additional studies (Vogel et al., 2009; Tomita et al., 2018). A filtering approach, based on dual PPG signal acquisition in both ears and asynchronous noise removal, was proposed by Tomita et al. and tested under various movement and exercise conditions (Tomita et al., 2018). The approach demonstrated higher performance (bias within ±5 bpm in 95% of measurements) than accelerometer-based filtering, at the cost of doubling the number of sensors and signals to be acquired. Vogel et al. compared the performance of time-domain and frequency-domain algorithms in estimating PR from PPG signals acquired by an in-ear MORES sensor (Vogel et al., 2009), connected with a small body box containing the signal acquisition and communication circuitry and granting hours of monitoring. The authors showed better performance of the frequency-domain method when working on either IR and red-light PPG signals, with the highest performance obtained using IR signals (bias of −0.59 ± 1.33 bpm with respect to a finger sensor).

### Measurement of Oxygen Saturation

#### Introduction

SpO_2_ is the physiological parameter which tracks the amount of oxygen dissolved in arterial blood or transported oxygen bound to hemoglobin, as indirectly measured at suitable peripheral sites (i.e., finger, toe, forehead, and ear) by pulse oximetry. Since the brain, as well as the muscles, requires a constant oxygen supply to support the high metabolic rate production necessary to remain electrically active (Williams et al., 2019), SpO_2_ monitoring is of relevance to detect possible risk conditions not only in clinical settings, but also in physiological studies, sport science, and occupational medicine (Costello et al., 2020; Pham et al., 2020; Stensrud et al., 2020). A reduction in oxygen availability, as experienced at high altitudes or in particular challenging situations, can have a detrimental effect on brain and muscles function, inducing performance decline and increasing the risk of errors and injuries (Hoiland et al., 2016). The parameter is also crucial to evaluate adaptation processes and performance when exercising or training at altitude or in hyperbaric conditions (Chapman, 2013). In high-risk environments for workers (firefighters or military personnel) and athletes, SpO_2_ information may help to prevent injuries, such as exertional heat illness (Pham et al., 2020). Advances in medical instrumentation and considerable improvements in optoelectronics have made pulse oximetry integrable in wearable devices (Foo et al., 2013), opening the possibility of long-term oxygen monitoring in the mentioned settings. On the other hand, in the presence of local or systemic vasoconstriction during sport activities or cold environmental conditions, poor peripheral perfusion at the wrist, finger, and toe, may make these sites inappropriate to accurately estimate SpO_2_ (Budidha and Kyriacou, 2018). The ear canal may represent a more reliable site for the assessment of SpO_2_, since it is less subject to vasoconstriction and motion artifacts and it guarantees suitable blood flux and PPG signal quality (Budidha and Kyriacou, 2014).

#### Results and Discussion

The three studies, which analyzed pulse oximetry measurements from the ear canal, are summarized in Table 4. All the studies analyzed prototype devices (Venema et al., 2012; Budidha and Kyriacou, 2014, 2018) that applied dual wavelength emission in reflection configuration. Performance was tested under various conditions. In two studies measurements were performed under challenging conditions inducing vasoconstriction, such as cold immersion test (Budidha and Kyriacou, 2014) and cold-exposure (Budidha and Kyriacou, 2018), while in the third study the device was calibrated under hypoxic conditions (Venema et al., 2012). The two studies performing vasoconstrictive maneuvers consistently showed that SpO_2_ measurements performed in the ear canal were minimally affected by vasoconstriction, while reference measurements performed on the fingertip were significantly impaired by the reduced blood flow. In particular, the amplitude of both red and IR PPG waveforms underwent just minor changes (+2.5 and −1.2%, respectively) after ice water immersion of the right hand with respect to baseline recordings, while the amplitude of both right and left index finger PPG signals resulted almost halved (Budidha and Kyriacou, 2014). Similar, in subjects undergoing artificially-induced hypothermia by means of cold exposure at 10°C, the normalized pulse amplitude of red and IR light PPGs presented a maximal decrease of 13% for the ear sensor with respect to a drop of >80% for the finger sensor. The decrease in signal amplitude in the finger sensor resulted in measurement failure (i.e., SpO_2_ < 90%) in one third of the subjects, while failure in ear measurement was obtained only in one subject out of 15 (Budidha and Kyriacou, 2018). These results supported the reliability of PPG signal quality from the ear canal during vasoconstriction, but they did not provide information on the actual accuracy of the estimated SpO_2_ values with respect to gold standard values. A single study examined this aspect and evaluated the performance of ear-in pulse oximeters in comparison with blood gas analysis under realistic clinical conditions with various levels of hypoxia (Venema et al., 2012). Specifically, SpO_2_ was measured by a PPG sensor embedded in an individually-customized ear mold for measurement in the ear canal, in healthy subjects wearing a breathing mask that regulated the blood oxygen level of the inspired air. During the tests blood was saturated in steps from 100% to 70–77% and blood samples from the radial artery were taken at each step to perform reference blood gas analysis. The study showed good correlation (*r*^2^ = 0.96) between absorbance rates and corresponding reference SpO_2_ values at the individual level (Venema et al., 2012). However, the global calibration curve obtained from all measurements showed unacceptable accuracy, due to the presence of offsets between individual calibration curves. Intra-individual variability among calibration curves enabled the estimation of SpO_2_ only in relative terms. Absolute reliable in-ear oxygen saturation measurements could be obtained in the study using an individual single-point calibration (i.e., performing an initial SpO_2_ measurement with a reference device). Alternative compensation strategies may rely on the use of a third wavelength to compensate for the offset without the necessity of single-point calibration (Venema et al., 2012).

## Limitations and Future Developments

The large set of research studies presented in this review proves the wide interest for hearables as suitable instruments for physiological parameters monitoring, potentially able to satisfy commercial and research-oriented markets. The data presented in this review suggest the capability of the devices to provide accurate measurements in multiple conditions, but point out the necessity of further improvements to guarantee reliability of measurements in more challenging scenarios, such as during intense exercise, under extreme climatic conditions, and/or dynamic conditions. These aspects are of primary importance to grant applicability in sport science and physiological research, as well as in emergency and occupational care. In general, a certain degree of variability in accuracy and performance was observed among studies. Although devices were often constructed based on similar principles, proprietary differences in technology and algorithms for signal processing may partially explain these differences. More importantly, heterogeneity in protocols (e.g., differences in the type, intensity, and duration of activities performed during the validation) and settings (e.g., simulation of extreme environments), as well as in the type of reference measures, were present among studies which may further hinder comparisons and a pooled evaluation of accuracy. Moreover, mismatch between requested standards and in-field accuracy may further explain differences among studies, which fosters further debate regarding the accuracy, validity, and reliability of in-ear measurements methods (Ring et al., 2010; Sund-Levander and Grodzinsky, 2013). Increasing the accuracy of measurements may rely on the improvement of signal/data acquisition and device stability, potentially through the implementation of new materials, as exemplified by graphene-coated T sensors (Chaglla et al., 2018). Probe development for T measurement should pursue personalization to adapt the device to the specific anatomy of each user (Muir et al., 2001; Venema et al., 2012; Ota et al., 2017), allowing a better insulation from external conditions (Teunissen et al., 2011; Strapazzon et al., 2015). For PPG-based measurements, accuracy may be improved by optimizing sensor design and wavelength [e.g., optimal LED-photodiode distance, shunt-light reduction (Buschmann and Huang, 2010)], but also by developing suitable signal processing and advanced algorithms for noise removal (Poh et al., 2010; Poh and Kittler, 2012; Tomita et al., 2018). Motion artifact removal and noise cancellation techniques are a central area of interest for the improvement of PPG-based monitoring devices for either PR and SpO_2_ measurement, and adaptive noise cancellation using integrated accelerometers as reference is considered a promising technique (Poh et al., 2010). Calibration issues and model optimization on larger populations under different conditions are relevant for obtaining reliable SpO_2_ measurements (Venema et al., 2012), as well as energy and metabolic parameters from PR values and accelerometric information (Leboeuf et al., 2014; Boudreaux et al., 2018). The use of advanced post-processing algorithms, allowing the extraction of respiratory traces and HR variability parameters from in-ear signals, may further extend the set of monitored physiological parameters and should be analyzed in future studies (Poh and Kittler, 2012). In addition, the possibility of measuring an ear-ECG instead of simply measuring a pulsatile signal may enable the identification and examination of heart conditions, such myocardial infarction or arrhythmias, which may be helpful for emergency medicine, sport medicine, and occupational medicine settings (von Rosenberg et al., 2017).

Further research is also needed to address limitations evidenced by the present review. The literature on in-ear SpO_2_ measurements is sparse and focused on signal quality issues. Calibration needs to be further addressed and algorithms need optimization on large study groups in comparison with gold-standard references (Venema et al., 2012). The growing demands for remote personal health monitoring for healthy geriatrics in residential homes (Foo et al., 2013), further stimulated by the recent COVID-19 pandemic, has promoted further research on in-ear SpO_2_ measurement for long-term monitoring. Recent data about a full in-ear SpO_2_ monitor, tested in resting subjects during a sequence of normal breathing and breath-holds (Davies et al., 2020), showed good agreement between in-ear and finger measurements, with a root mean square error of 1.47% and just slight overestimation of SpO_2_ by the ear device. Of note, the device showed a faster response to track saturation changes induced by the breath hold protocol than the finger sensor (Davies et al., 2020). As concerns T, tympanic measurements have been shown to track body T and potentially brain T, but this assumption should be considered with caution. Despite the shared vasculature with carotid artery, branches of the external carotid artery could introduce variability associated to the size, origin, and therefore relative flow through each component artery (McCarthy and Heusch, 2006). These aspects—together with biasing factors related to environmental conditions, operators training, in-ear local phenomena, and adequate maintenance of the probes—may represent further sources of variability (Bridges and Thomas, 2009; Ring et al., 2010; Sund-Levander and Grodzinsky, 2013) with impact on T measurements in different physiological and pathophysiological conditions. Thus, the actual reliability and range of validity of in-ear physiological parameters need further tests, especially in challenging environments and in real patients. As well, technical challenges in terms of power, cost, size, weight, functionality, and packaging needs further efforts in view of long-term applications and more challenging settings. Such efforts will be potentially rewarded, since in-ear technology is integrable with smart devices, and thus it may be packaged and sold with smartphones as audio earbuds in large volumes of units per year (Leboeuf et al., 2014). Thanks to their unobstrusivity and comfort, hearables may reach a larger consumer audience, with potential benefit in the promotion of healthy lifestyles and preventive healthcare, and they may offer a novel non-invasive site for monitoring workers and patients in out-of-hospital settings.

## Conclusive Remarks

The accumulating evidence presented in this review supports the promise of in-ear sensors as an innovative and unobtrusive way to monitor vitals in daily-life and during physical activity, although applications in more challenging environmental scenarios and intense exercise settings require further improvements in terms of accuracy. Further research addressing physiological and technical aspects is strongly encouraged to better understand the peculiar anatomical and vascular features of this unique body site in order to ameliorate measurement accuracy and device response in different contexts.

## Data Availability Statement

All relevant data is contained within the article.

## Author Contributions

MM and AM designing the study, performing the literature search, and drafting the manuscript. GS designed the study and critically revised the manuscript for important intellectual content. All authors contributed to manuscript revision, read, and approved the submitted version.

## Conflict of Interest

The authors declare that the research was conducted in the absence of any commercial or financial relationships that could be construed as a potential conflict of interest.
